# An Important Indian Traditional Drug of Ayurveda Jatamansi and Its Substitute Bhootkeshi: Chemical Profiling and Antioxidant Activity

**DOI:** 10.1155/2013/142517

**Published:** 2013-03-21

**Authors:** Madan Mohan Pandey, Antariksha Katara, Garima Pandey, Subha Rastogi, A. K. S. Rawat

**Affiliations:** Pharmacognosy & Ethnopharmacology Division, CSIR- National Botanical Research Institute, Lucknow 226001, India

## Abstract

*Nardostachys jatamansi* DC. and *Selinum vaginatum* (Edgew) Cl. are two endemic high altitude Indian medicinal plants that have been traditionally known as “Jatamansi” and “Bhootkeshi,” respectively. These are used in various traditional herbal formulations and nutraceuticals, as well as to treat neurological disorders like epilepsy, hysteria, syncope, convulsions, and mental weakness. They resemble each other in their external morphological characters and characteristic odour, so their roots are often confused with each other. Since free radicals have been implicated in the pathogenesis of a considerable range of neurological disorders, including seizures and epilepsy, analysis of these two important medicinal plants was carried out based on their antioxidant activities and phenolic profiles. *N. jatamansi* expressed better antioxidant activity with both DPPH and TAC methods. Strong correlation was seen between TPC and antioxidant activities. Phenolic compounds such as chlorogenic acid, ferulic acid, protocatechuic acid, and syringic acid were analyzed qualitatively and quantitatively in the methanol extracts of *N. jatamansi* and *S. vaginatum* by HPLC. *N. jatamansi* was found to contain only protocatechuic and syringic acids while chlorogenic and ferulic acids were present only in *S. vaginatum*. The studies suggest that both of the plants exhibit distinctive properties and that their similar therapeutic uses may be dependent on synergistic effects exhibited by the different compounds present in them.

## 1. Introduction

*Nardostachys jatamansi* DC. (Valerianaceae) and *Selinum vaginatum* C.B. Clarke (Umbelliferae) are two important indigenous drugs found in Himalayan region. The roots and the rhizomes of *N. jatamansi,* as mentioned in Ayurveda, have been used in various herbal formulations including dietary supplements. This important traditional drug is also used to treat epilepsy, hysteria, syncope, convulsions, and mental weakness. The decoction of the drug is also used in neurological disorders, insomnia, and disorders of cardiovascular system. It has been reported to exhibit antidepressant, anticonvulsant and antiarrhythmic activities as well as to possess antioxidant and lipid peroxidation activities [1, 2]. The sesquiterpenes (jatamansic acid, jatamansone), lignans, and neolignans are reported to be present in the roots of this plant. *S. vaginatum* roots are also used as a nervine sedative and considered useful in hysteria. Its oil possesses sedative, analgesic, and hypotensive properties. The roots of *S. vaginatum* have yielded several coumarins, namely selinidin, selinone, angelisin, anomalin, isopteryxin, orosenol, lomantin, and vaginidin. The roots of *N. jatamansi* (commonly known as “Jatamansi”) and *S. vaginatum* (Bhootkeshi) are often confused with each other. Due to the resemblance in between external morphological characters and characteristic odour the roots of *S. vaginatum* are being used as a substitute for *N. jatamansi *in the Indian herbal drug market [3, 4].

Neuronal hyperexcitability and excessive production of free radicals have been implicated in the pathogenesis of a considerable range of neurological disorders, including seizures and epilepsy. Epilepsy is a chronic, dynamic neurological disorder associated with ongoing neuronal damage, particularly when uncontrolled. Oxidative injury may play a role in the initiation and progression of epilepsy. The large lipid content of myelin sheaths and the high rate of brain oxidative metabolism coupled with the low antioxidant defenses make the brain highly vulnerable to free radical damage [5, 6]. Thus, it appears that free radicals may be responsible for the development of convulsions. The increased susceptibility of the brain to oxidative damage and experimental and clinical data suggests a putative role of oxidative stress in the pathophysiology of certain seizure types. The prooxidant/antioxidant balance is not only modulated by seizures per se but also by antiepileptic drugs. The ability of antioxidants for reducing the seizure manifestations and the accompanying biochemical changes (i.e., markers of oxidative stress) further supports a role of free radicals in seizures and highlights a possible role of antioxidants as adjuncts to antiepileptic drugs for better seizure control.

Since both of these plants, that is, *N. jatamansi* and *S. vaginatum,* have been used traditionally for the treatment of epilepsy, seizures, hysteria, and so forth and because it has been well established that oxidative stress may be both an important cause and a consequence of seizures, it can be assumed that the antioxidant compounds present in these species may be responsible for their therapeutic uses. Phenolic acids are powerful antioxidants and have been reported to demonstrate several biological activities including antibacterial, antiviral, anticarcinogenic, anti-inflammatory and vasodilatory actions [7, 8]. To the best of our knowledge there have been no reports on the phenolic antioxidant components of both these plants. The present studies were thus carried out to evaluate their total phenolic content and antioxidant activities and to identify and quantify the major phenolic constituents present in them.

## 2. Materials and Methods

### 2.1. Standards and Reagents

Chlorogenic acid, ferulic acid, protocatechuic acid, syringic acid, rutin, and quercetin were obtained from Sigma-Aldrich (St. Louis, MO, USA). HPLC-grade acetonitrile, methanol, water, and phosphoric acid were obtained from Merck (Darmstadt, Germany). All chemicals and solvents were purchased from Merck Chemicals (Mumbai, India). Whatman (Florham Park, NJ, USA) No. 1 filter paper was used for filtration of the samples.

### 2.2. Sample Collection and Preparation of Extracts

The roots of *Nardostachys jatamansi* and *Selinum vaginatum* were collected from Milam region (Uttarakhand) and Rohtang (Himachal Pradesh), India, in the months of September and October 2008, respectively. They were authenticated by Dr. A. K. S. Rawat, Head of Pharmacognosy & Ethnopharmacology Division, CSIR-NBRI, by comparison with authentic herbarium specimens and samples and deposited (Voucher specimen numbers 227331 and 227332) in the departmental herbal drug museum for future reference. A known amount of each plant material (100 g each) was kept in an oven at 40°C for drying to constant weight. Air-dried (40–50°C) roots (50 g each) were powdered and extracted with warm methanol (3 × 100 mL) by stirring on a magnetic stirrer for 30 min each time. The extracts were combined, filtered through 45 *μ*m filter paper, and concentrated under vacuum to obtain the dry extracts. These methanolic extracts were stored at 5°C till further use. 

### 2.3. Determination of Total Phenolic Content

The total phenolic content (TPC) was determined using a modified colorimetric Folin-Ciocalteu method [9]. Briefly, 0.5 mL of deionized water and 0.125 mL of different extracts of known dilution (1 mg/mL) were added to reaction vial. Folin-Ciocalteu reagent (0.125 mL) was added to the solutions and allowed to react for 6 min. Then, 1.25 mL of 7% sodium carbonate solution was added to each reaction vial and the mixture was diluted to 3 mL with deionized water. The color was allowed to develop at room temperature and the final absorbance was read at 760 nm using a Thermo UV1 spectrophotometer after 90 min. A calibration curve was prepared using different concentrations of standard gallic acid solutions, each time an analysis was run. Total phenolic content in the samples was calculated from the standard calibration curve and the results were expressed as mg of gallic acid equivalents per gram (mg GAE/g) of dry extract. Each sample was measured in triplicate and the mean was taken.

### 2.4. DPPH Radical Scavenging Activity

2,2-Diphenyl-1-picrylhydrazyl (DPPH) radical scavenging activities of the extracts were investigated according to a previously reported method [10] with minor modifications. Briefly, to a methanolic solution of DPPH (100 mM, 2 mL), 2.0 mL of test sample dissolved in methanol was added at different concentrations (40–200 *μ*g/mL). Equal amount of methanol was added to the control. Absorbance was recorded at 517 nm at 5, 15 and 30 min. The scavenging activity was calculated using the formula of scavenging activity (%) = [(A_517_ of control−A_517_ of sample)/A_517_ of control] × 100. Ascorbic acid was used as a standard.

### 2.5. Total Antioxidant Capacity

The antioxidant capacity of the extract was measured spectrophotometrically using a phosphomolybdenum method [11], based on the reduction of Mo(VI) to Mo(V) by the sample analyte and the subsequent formation of specific green phosphate/Mo(V) compounds. 0.1 mL of the sample (40–300 *μ*g) solution was combined in an eppendorf tube with 1.0 mL of reagent solution (0.6 M sulfuric acid, 28 mM sodium phosphate, and 4 mM ammonium molybdate). The tubes were capped and incubated in a thermal block at 95°C for 90 min. After cooling to room temperature, the absorbance of the aqueous solution of each was measured at 695 nm against a blank. A typical blank solution contained 3.0 mL of reagent solution, and the appropriate volume of methanol was used for the dissolution of the samples and it was incubated under the same conditions as the rest of the samples. Ascorbic acid was used as the standard, and the total antioxidant capacity was expressed as equivalents of ascorbic acid.

### 2.6. HPLC Analysis

HPLC analyses were performed on a liquid chromatography system consisting of Waters (Milford, MA, USA) model 515 pumps and equipped with an online degasser, a Waters PCM (Pump Control Module), a Rheodyne 7725 injection valve furnished with a 20 **μ**L loop, a Waters 2996 photodiode array detector (PDA), and Waters Empower software. Each analysis was repeated three times, and the respective retention times were averaged. HPLC conditions: Purospherstar RP-8 column (5 **μ**m, 4.6 × 250 mm; Merck), guard column (4.6 × 40 mm) packed with the same material; solvent system: solvent A-water: phosphoric acid (99.7 : 0.3 v/v), solvent B-acetonitrile : water  : phosphoric acid (79.7 : 20 : 0.3 v/v); gradient 0–5 min with 88–85% A, 5-6 min with 85–82% A, 6–9.5 min with 82–75% A, 9.5–10.5 min with 75–74% A, 10.5–12 min with 74–73% A and 12–20 min with 73–70% A, 20–30 min with 70–30% A, and isocratic from 30 to 35 min with 30% A; flow rate: 0.8 mL/min; column temperature: 30°C; injection volume: 10 *μ*L; standard concentration: 0.1 mg/mL; sample concentration: 50 mg/mL; PDA detection: 280 nm, spectra 200–600 nm. 

## 3. Results

### 3.1. Determination of Total Phenolic Content

The methanolic extracts of *N. jatamansi* and *S. vaginatum* showed significant amount of phenolic content. The methanolic extracts of the roots of *N. jatamansi* contained 39.54 mg GAE g^−1^ which was nearly 1.7 times the TPC of the methanolic extract of the roots of *S. vaginatum* (22.74 mg GAE g^−1^) (Table 1). 

### 3.2. DPPH Radical Scavenging Activity

**Figure 1:**
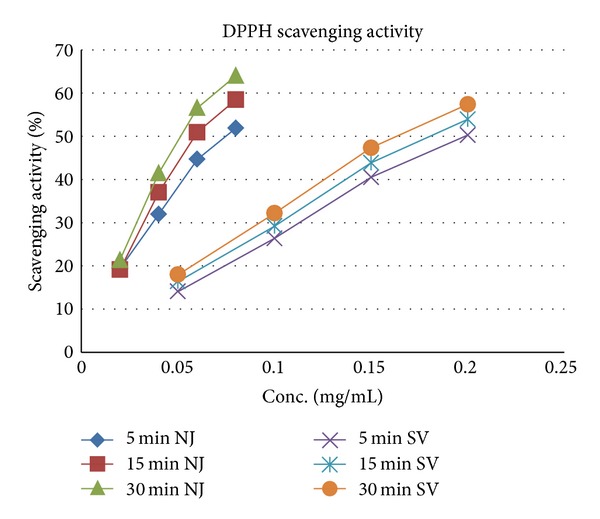
DPPH radical scavenging activity of *N. Jatamansi* and *S. vaginatum. *

The DPPH radical scavenging activities of the methanolic extracts of *N. jatamansi* and *S. vaginatum* are given in Table 2. DPPH radicals react with suitable reducing agents losing colur stoichinometrically with the number of electrons consumed which is measured spectrophotometrically at 517 nm. As shown in Figure 1, *N. jatamansi* extract strongly scavenged DPPH radicals with the IC_50_ being 52 *μ*g/mL as compared to 165 *μ*g/mL for *S. vaginatum*. The scavenging was found to be concentration dependent (*R* ^2^ > 0.96) and time dependent (*R* ^2^ > 0.97) (Figure 1) with the highest activity being observed at 30 mins, where at a concentration of 80 *μ*g/mL *N. jatamansi* showed 64.1% activity as compared to 57.48% exhibited by *S. vaginatum* at a concentration of 200 *μ*g/mL. Standard ascorbic acid was found to exhibit 83.19% activity at 5 *μ*g/mL concentration. 

### 3.3. Total Antioxidant Capacity

**Figure 2:**
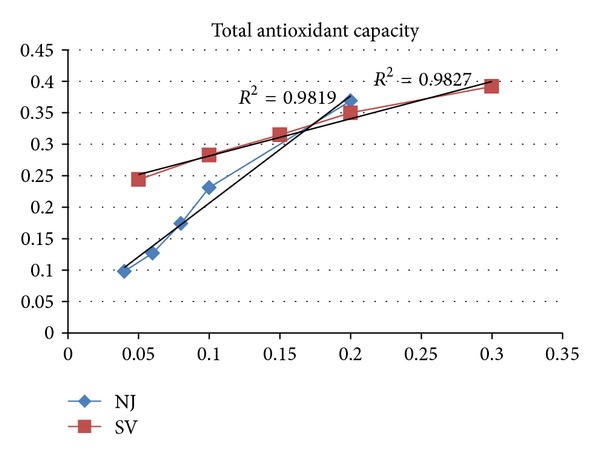
Spectrophotometrically measured phosphomolybdenum complex as a function of concentration of *N. Jatamansi* and *S. vaginatum. *

The total antioxidant capacity of the extract was calculated based on the formation of the phosphomolybdenum complex which was measured spectrophotometrically at 695 nm. A direct correlation was found to exist between the concentration of the extract used and the spectrophotometrically measured phosphomolybdenum complex (*R* ^2^ > 0.98) (Figure 2). The total antioxidant capacity of the *N. jatamansi* and *S. vaginatum* extracts was found to be 151 and 143 *μ*mol ascorbic acid equivalent/g, respectively (Table 2). 

### 3.4. Correlation between Antioxidant Activities and Total Phenolic Content

Correlation studies were also carried out between antioxidant activities and total phenolic content (Table 3). Based on correlation analysis, DPPH radical scavenging activities of both *N. jatamansi* and *S. vaginatum* were strongly correlated with total phenolics content of methanolic contents of the extract (*N. jatamansi*, *r* = 0.9808; *S. vaginatum*, *r* = 0.9965). Similarly the spectrophotometrically measured phosphomolybdenum complex was also strongly correlated with total phenolics content of these extracts (*N. jatamansi*, *r* = 0.9909; *S. vaginatum*, *r* = 0.9897).

### 3.5. HPLC Analysis

**Figure 3:**
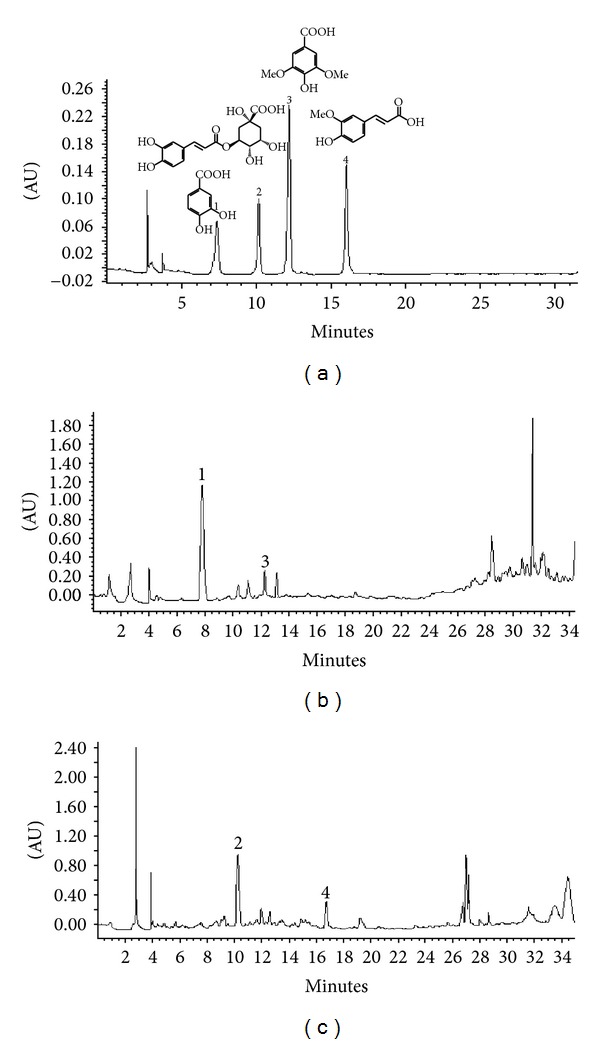
HPLC profile of phenolic acids. (a) Standard mixture (1-protocatechuic acid, 2-chlorogenic acid, 3-syringic acid, 4-ferulic acid); (b) *N. Jatamansi* methanolic extract; (c) *S. vaginatum* methanolilc extract.

In order to determine the phenolic acid composition of *N. jatamansi* and *S. vaginatum*, their methanolic extracts were subjected to HPLC analysis under the conditions described above. Some well-known antioxidant polyphenolics, namely, chlorogenic acid, ferulic acid, protocatechuic acid, syringic acid, rutin and quercetin, were analyzed. These compounds have been of interest due to their many potential health benefits. The HPLC studies showed that out of the compounds analysed, only the phenolic acids (chlorogenic acid, ferulic acid, protocatechuic acid, and syringic acid) were found to be present either in *N. jatamansi* or *S. vaginatum*. Rutin and quercetin were not detected.  Figure 3 shows the HPLC chromatograms of the methanolic extracts of *N. jatamansi* and *S. vaginatum* monitored at 280 nm. Results of the HPLC analysis are presented in Table 1. Our observations with *N. jatamansi* and *S. vaginatum* using the four known phenolic acids indicated that there was a marked specificity to the type of phenolic acid present—*N. jatamansi* contained only benzoic acid derivatives (protocatechuic acid and syringic acid) while hydroxycinnamic acid derivatives (chlorogenic acid and ferulic acid) were present only in the methanolic extract of *S. vaginatum*. Chlorogenic acid and protocatechuic acid were present in substantial quantities in *S. vaginatum* and *N. jatamansi,* respectively.

## 4. Discussion

In the present investigation, the antioxidant properties and phenolic content of two important indigenous drugs of India, *Nardostachys jatamansi* and *Selinum vaginatum*, used in neurological disorders like epilepsy, hysteria, syncope, convulsions, and mental weakness were studied. A number of studies suggest that oxidative stress plays an important role in the etiology of epilepsy and other neurological disorders. Studies demonstrate that prolonged seizures acutely result in oxidative damage to lipids, DNA, and susceptible proteins. Such mechanisms (e.g., oxidative stress) could independently contribute to the disease progression in addition to serving as processes that underlie neuronal injury. It has been observed that compounds with antioxidant properties (superoxide dismutase (SOD) mimetics, vitamin C, spin traps, and melatonin) prevent seizure-induced pathology [12–16]. Newer antiepileptic drugs such as zonisamide possess antioxidant properties [17]. Thus therapies aimed at reducing oxidative stress may ameliorate tissue damage and favorably alter the clinical course.

The main purpose of this study was to evaluate these two endemic high altitude Indian medicinal plants based on their phenolic profiles and antioxidant studies. Since phenolic acids are well known to be potent antioxidants, the phenolic antioxidant components present in *N. jatamansi* and *S. vaginatum *were identified. Marked difference in their phenolic profiles was observed with *N. jatamansi* containing only protocatechuic and syringic acids while chlorogenic and ferulic acids were present only in the methanolic extract of *S. vaginatum*, indicating that only benzoic acid derivatives and hydroxycinnamic acid derivatives were present in *N. jatamansi* and *S. vaginatum,* respectively. However, a more detailed study, employing many more derivatives belonging to the two groups of phenolic acids, needs to be carried out before such a conclusion is finally drawn. Secondly, both plants exhibited significant variation in their TPC and antioxidant properties. *N. jatamansi* exhibited a higher TPC as well as better antioxidant activity than *S. vaginatum*. The results demonstrated that although the roots of *N. jatamansi* (commonly known as Bhootjataa) and *S. vaginatum* (Bhootkeshi) resemble each other in their external morphological characters and characteristic odour and are often confused with each other, *S. vaginatum* roots should not be used as a substitute for *N. jatamansi*. But, since both of these plants, that is, *N. jatamansi* and *S. vaginatum,* have been used traditionally for the treatment of epilepsy, seizures, hysteria, mental weakness, and so forth and because it has been well established that oxidative stress may be both an important cause and a consequence of seizures, it can be assumed that the activity exhibited by them is dependent on synergistic effects exhibited by the different compounds present in these species that may be responsible for their therapeutic uses.

## Figures and Tables

**Table 1:** Total phenolic content and major phenolic acids identified in methanolic extract of *N. jatamansi* and *S. vaginatum. *

Name	TPC (gallic acid equ) mg/g	Phenolic compounds identified by HPLC (mg/100 g extract)			
		Protocatechuic acid (1)	Chlorogenic acid (2)	Syringic acid (3)	Ferulic acid (4)
*N. jatamansi *	39.544 ± 2.16	596.5 ± 1.61	—	68.5 ± 0.22	—
*S. vaginatum *	22.744 ± 1.23	—	1054.3 ± 2.86	—	61.9 ± 1.08

Values are mean ± SD (*n* = 3).

**Table 2:** Antioxidant effect on free DPPH radicals and total antioxidant capacity.

Plant name	DPPH radical scavenging ability (IC_50_, *μ*g/mL)	Total antioxidant capacity (*μ*mol AA/g extract)
*N. jatamansi *	50	151
*S. vaginatum *	165	143
Ascorbic acid (AA)	2.3	—

**Table 3:** Correlation between bioactivities and total phenolic content.

Activities	Correlation coefficient (*r*)	
	*N. jatamansi *	*S. vaginatum *
DPPH radical scavenging activity (% inhibition)	0.9808	0.9965
Total antioxidant capacity		
(i) Absorption for phosphomolybdenum complex formed	0.9909	0.9897
(ii) Ascorbic acid equivalent (AAE)	0.9824	0.9897
